# Camel pastoralists’ perceptions of udder health: results from a qualitative interview study in Northern Kenya

**DOI:** 10.1186/s12917-025-04723-x

**Published:** 2025-04-14

**Authors:** Dinah Seligsohn, Khalif A. Abey, Erika Chenais

**Affiliations:** 1 Department of Animal Health and Antibiotic Strategies, Swedish Veterinary Agency, Uppsala, SE-75189 Sweden; 2 Department of Disease Control, School of Veterinary Medicine, The University of Zambia, P.O Box 32379, Lusaka, 10101 Zambia; 3 Kenya Camel Association, 10th Floor, Hill Plaza, Upperhill, Nairobi, Kenya; 4 Department of Epidemiology, Surveillance and Risk Assessment, Swedish Veterinary Agency, Uppsala, SE-75189 Sweden; 5 Department of Animal Biosciences, Swedish University of Agricultural Sciences, Uppsala, SE-75007 Sweden

**Keywords:** Camel milk, Mastitis, Focus group discussions, Pastoralists, Preventative measures

## Abstract

**Background:**

In the Horn of Africa, pastoralists depend on livestock for food security. Camels play a pivotal role, providing both milk and meat to these communities. Camel milk production is hampered by multiple factors, one of the main issues being diseases, in particular udder health issues such as mastitis; inflammation of the udder. This study aimed to enhance understanding of camel keeping pastoralists’ perceptions of mastitis and attitudes toward its control in in camels. The study was carried out in Isiolo County in Kenya. Data collection involved focus group discussion (FGDs) with camel owners, herders and actors involved in the camel milk trade. Participants predominantly belonged to the Borana, Somali and Sakuye communities. The data pertaining to perceptions of udder health problems and preventive measures was thematically analysed, using an inductive approach.

**Results:**

Four themes were identified regarding pastoralists perceptions of udder health; (i) Importance of udder health; (ii) Economy; (iii) Herders’ responsibilities and (vi) Udder health strategies. The results showed that camel pastoralists were skilled at detecting clinical mastitis and had developed strategies to maintain good udder health, such as adhering to a milking order, culling camels with faulty udders and applying treatment. The use of traditional knowledge in parallel with modern medical knowledge resulted in a multitude of treatment strategies without any standardised treatment routines. Awareness of subclinical mastitis, that is mastitis without any clinical signs, was very low and its detrimental effects of mastitis on milk production were poorly understood. Participants were largely positive to interventions that were sustainable, feasible, effective and accessible, such as using California Mastitis Test (CMT) and hand disinfectant.

**Conclusions:**

Strategic milk hygiene work including selected interventions accompanied by targeted educational efforts and driven by economic incentives, could form the basis for future preventative work. This will need to be evaluated in the local context.

**Supplementary Information:**

The online version contains supplementary material available at 10.1186/s12917-025-04723-x.

## Introduction

Livestock keeping is a cornerstone for pastoral and agro-pastoral livelihoods in Kenya, providing food security and playing an important economic, social and cultural role [1]. In arid and semi-arid lands, camels are the most important production animals because of their resilience and ability to maintain milk production in a hostile environment. Camel milk is an important part of the diet and can contribute up to 40% of pastoralists’ daily calorie intake [2]. Mastitis (clinical and subclinical (SCM)) is a common constraint among camels kept for milk production with implications for milk production, milk safety, animal welfare and household economy [3]. In a study from Kenya, the post-harvest losses due to mastitis were estimated to be 76% of total milk production [4]. Studies in pastoralist areas in Kenya indicate that mastitis is a regular occurrence among dairy camels, revealing animal level prevalence between 46% and 66% [3, 5]. Clinical mastitis is usually recognised by pastoralists [6–9]. In a study of camel pastoralists in Somalia, mastitis was listed as the main constraint for milk production [10] and it has also been reported as a common problem among several other camel-keeping communities [4, 5]. Since SCM cannot be detected without assessing the levels of somatic cells or other changes in milk composition which requires testing of the milk, there is generally a lack of awareness of SCM among pastoralist groups [3].

Perceptions of mastitis and the underlying causative agents vary across geographical regions. Among Senegalese pastoralists, mastitis is thought to be caused by the boiling of milk [11]; according to the beliefs of the Sahrawi pastoralists in Western Sahara, mastitis is caused by high stocking densities or retention of milk; while mastitis is generally attributed to “the evil eye” among many pastoralist groups in the Horn of Africa and Middle East [6, 12, 13]. Traditional remedies for mastitis include herbal medicine [13–15], abstaining from milking the affected quarter, removal of ticks and cauterisation of the udder [7, 15–18]. Moreover, with the increasing availability of modern veterinary drugs to camel pastoralists, treatment with antibiotics is now common [19].

Historically, most studies on mastitis in pastoralist camels have focused on investigating the prevalence of infectious agents or assessing risk factors. To the knowledge of the authors, no studies have investigated the social and cultural aspects of how pastoralist camel keepers perceive mastitis and manage udder health. Such knowledge is needed in order to find treatments, prevention or control measures for mastitis that are feasible and acceptable and thus possible to implement in the pastoralist setting. This includes studying factors affecting camel keepers’ possibilities to implement different measures and reasons behind compliance versus non-compliance. Furthermore, research suggests that in order to achieve sustainable change, communities need to be engaged in the entire process from defining the problem to finding the solutions [20–22]. Participatory methods have been recommended for this purpose [23–25]. The aims of the study were to increase the understanding about camel keeping pastoralists’ perceptions and knowledge of mastitis and their perceptions and attitudes to mastitis control in camels in Northern Kenya. To reach these aims, a participatory approach using qualitative research methods allowing for a focus on individuals’ descriptions of their own experiences was used.

## Materials and methods

This study reports of data concerning mastitis and udder health, results from the same field study but pertaining to other challenges will be reported in separate articles.

### Study area and study design

This qualitative interview study using focus group discussions (FGDs) was conducted in two locations in Isiolo County, Kenya; Isiolo town and Kula Mawe, in November and December 2022. This time represents the end of the dry season and the early wet season. Isiolo County borders Marsabit, Wajir, Garissa, Tana River, Meru, Laikipia and Samburu counties. The county is largely classified as very arid, the annual rainfall measures 150–250 mm [26]. Camel pastoralism is well adapted to these circumstances and is a common livelihood in the region. The estimated camel population in the county is 162,516 heads [27]. Camel-keeping households in the area include ethnic Somali, Borana, Sakuye, Garre, Turkana and Samburu. Ongoing climate change characterized by prolonged droughts and erratic rainfall, has necessitated livestock diversification prompting more people to acquire camels to enhance resilience [28]. Isiolo town is an important hub for camel milk production in Northern Kenya, both with a developing peri-urban milk production but also as a bulking centre for milk distribution lines coming from other counties. The milk is bulked and transported to Nairobi and other final destinations. The largest milking cluster and the highest density of camels can be found in and around Isiolo Township, located in Isiolo Central division [29]. Two major women-led camel milk cooperatives dominate the market. The area has been subjected to numerous initiatives by local and international NGOs to promote camels as a means of livestock diversification and improved resilience among the traditionally cattle keeping Borana [30]. Isiolo also has a large population of Somalis, for decades engaged in the trading and handling of camel milk. Kula Mawe is a rural town/village of approximately 800 households, the population being predominantly Sakuyes and Borans. In Kula Mawe, a smaller milk cooperative has just started operating.

The study locations were purposively selected based on ongoing camel milk production and trade in the area and accessibility to relevant stakeholders in the camel milk value chain. Participants were purposively selected based on involvement in the camel milk trade and recruited by local mobilisers/key informants via the local milk cooperatives together with the facilitator and the translator. Initial instructions for recruitment were to organise groups of 3–9 persons separated according to gender (male/female) and occupation (camel owner/herder/milk seller). Keeping herders and owners separated was however advised against by key informants as this could create suspicion among the camel owners and was thus not done.

### Data collection

The research team consisted of a senior researcher (EC), a researcher (DS), a facilitator (KA) and a translator. FGDs were guided by a topic guide (Supplementary material 1), including open questions encompassing camel management, challenges and diseases, and perceptions of these. Both EC and DS are trained veterinarians, KA comes from a camel keeping background and the translator is an Animal Health Officer in the area. Both KA and the translator are proficient in the local language Oromo as well as in Swahili and in English. The topic guide had been translated jointly by KA and the translator from English to Oromo and Swahili. Prior to roll-out of the study the topic guide was tested on a pilot group consisting of three male herders/camel owners to assess relevance of the questions and length of the discussion and adjusted accordingly. The discussions were led by KA in in either Oromo or Swahili and simultaneously translated to English. The translations were recorded for back up but not transcribed *ad verbatim*. DS and EC took detailed notes of the translations and intervened with follow-up questions or for clarifications if necessary. At the end of each day the notes were merged to form a master note for each FGD. The discussions were opened by the facilitator introducing the research team, explaining the background of the study and informing the participants about the purpose and objectives. The participants were informed that participation was anonymous, voluntary, and that they could withdraw from study at any given moment. Written consent to participate, to be recorded and to be photographed was obtained from all participants before continuing the discussion. Participants were offered a snack during the discussion and compensated for their travel costs. Throughout the discussions, the participants were encouraged to share their views and ask questions to the research team and each other.

Following the initial information, participants were asked to introduce themselves by name and describe their involvement in the camel milk value chain. The discussions were continued by asking questions about camel keeping and perceptions on camel health. The participants were asked to describe the most common diseases in their herds and to rank these in order of importance. In addition to discussing diseases, participants were asked about strategies for keeping their camels healthy (these results will be presented in another publication). This was followed by a discussion more focused on udder health including preventative strategies, treatment of udder problems, causes and effects. After this, the facilitator presented a set of suggested interventions aimed at improving the udder health and the participants would discuss the perceived advantages and/or disadvantages with these tools. Finally, the participants were asked if there was anything they would like to discuss that had not already been voiced, and if they had any final questions to the research team.

### Data management and analysis

The analysis started in the field by discussing the findings with the facilitator, the translator and key informants. Before starting the post-field-analysis, DS listened through the recordings to validate the master notes. Following, the master notes were imported to NVivo software release 1.7.1 (QSR International, Australia) for qualitative analysis. DS performed the preliminary analysis, after which DS and EC iteratively reviewed the results, and finally all three authors reviewed and discussed the results. Based on the included topics and on the data content (thus determined partly in advance of data collection and partly during the analysis), the results were divided in three sections, and analysed using two different methods: (1) For data related to the topics belonging to clinical presentation of udder problems and causes of udder problems (topic 10, 11,13 in the topic guide, Supplementary material 1), content analysis was performed. These results are presented descriptively in the section “Categories, causes and clinical signs of mastitis”. (2) The topics discussing perceptions of udder problems (topic 9, 12, 14–17) and perceptions of possible interventions for preventing udder problems (topic 18–22) were subjected to thematic analysis, separately for the each of these two groups of topics. These results are presented in the sections “Perceptions of udder problems” and “Perceptions of measures for preventing udder problems” respectively. Thematic analysis was used to identify, analyse and interpret patterns of meaning (themes) within the data in each section. An inductive approach was applied, meaning that themes were formed based on the data itself. The thematic analysis started with repeated reading of the transcripts. Following, initial codes were developed and codes that belonged together were grouped to form sub-themes, and finally themes were formed to create a narrative around the sub-themes based on the ideas considered to best demonstrate the perceptions of the participants. The process was iterative and carried out until DS and EC determined that the themes identified from the data were sufficient for the study aims. Content analysis was done for the topics which didn’t include suitable, or deep enough, information to form such narratives, and represents a summary of the data content in those topics. To capture the differences in the data and highlight interesting findings, quotes have been included. As these quotes are not direct translations of what was said, but deriving from the notes, they should be seen as illustrations of the themes.

### Researcher reflexivity

This study was a collaboration between researchers from Sweden (DS and EC) and Kenya (KA). DS and EC are both Swedish veterinarians with experience from conducting research in Kenya and other low-income countries. KA is Kenyan, from a camel owning family and works daily with camel owners in Kenya for a national organisation based in Nairobi. KA have extensive local knowledge, knowledge about animal health in general and specifically concerning camels, as well as experience in participatory methods. DS did her PhD work on subclinical mastitis in pastoralist camels in Kenya, her work served as rationale for the current work. EC has experience with qualitative and participatory research in low-income countries including with pastoralists. DS’s prior knowledge and experience with camel mastitis inspired the content of the study whereas EC’s prior knowledge and experience mainly influenced the methodology. In the context of the study, both DS and EC can be considered as ‘outsiders’, whereas KA can be considered an ‘insider’ in most, but not all, instances. Being an outsider can make the asking of naïve question more acceptable than if they were asked by an insider and help in keeping an analytic distance to the research situation, but can also prevent deeper participation and understanding [31]. The ‘outsider’ positionality, and specifically not speaking the local language, made DS and EC dependent on KA and the interpreter for translation and for providing contextual understanding. Having to rely on translation is an important limiting factor in qualitative research, creating risks for loss of data quality and depth. Using simultaneous translation gave an opportunity to interact in the moment of the data collection, to some extent diminishing this limitation. Likewise, having KA and a skilled interpreter with local connections in the research team, and starting the data analysis already in the field, helped to establish report between DS and EC and the data.

## Results

In total, 13 FGDs were held, comprising 85 participants in groups of 3–12 people. In all, 46 women and 39 men participated in the FGDs, five groups consisted of only female participants, six with only men and two groups were mixed. Most of the participants were either camel owners or involved in camel milk trading (predominantly as a part of one of the milk-selling women cooperatives). Twelve participants were employed herders, but most of the camel owners also stated that they herded their own camels. Overall saturation, meaning that no new information relevant for answering the research questions came up in the discussion, was deemed to have been achieved after 10 FGDs. The results were divided in three sections, developed below: (1) categories, causes, and clinical signs of mastitis, (2) perceptions of udder problems, and (3) perceptions of measures for preventing udder problems.

### Categories, causes and clinical signs of mastitis

Clinical mastitis was recognised among the participants, and descriptions of the clinical presentation were extensive, complex and detailed (Table 1). Most participants categorised udder health problems according to three major syndromes: swelling of the udder, wounds on the udder or teats and bloody milk. The categories ‘swollen udder’ and ‘wounds on the udder/teats’ were not entirely separate and were sometimes mentioned together.

**Table 1:** Clinical signs associated with udder problems mentioned in 13 FGDs with camel pastoralists in Isiolo County, Kenya

Clinical signs	Number of groups mentioned in
Swelling of the udder	13
Changes in the milk (general)	13
Changes in the milk: pus	12
Changes in the milk: particles/clots	7
Changes in the milk: bloody milk	7
Changes in the milk: watery milk	1
Changes in the milk: abnormal milk	3
Painful udder	9
Heat around the udder	9
Aggressive behaviour during milking	9
Wounds around the teats/udder	6
Fever	4
Blockage of teats	3
Low milk production	2
Unpleasant odour from the milk	2
Smaller teat in the affected quarter	2
Reddish appearance on the udder	1
The camel is not eating	1
Unwilling to walk	1

When discussing the possible causes of udder health problems, participants offered a wide range of explanations and beliefs, often nested within each other, and intermingled. The classifications were sometimes difficult to separate from each other, and the three categories of udder problems presented here are thus not rigid. The following quote illustrates a differentiation between “swollen udder (Barar)” and “wounds on the udder (Arar)”, however not all groups made the same distinction:

*“Barar– swelling of the udder so teats become small*,* pus*,* difficult to milk*,* painful for the camel*,* sometimes blood in the milk*,* calves not allowed to suckle*,* camel becomes aggressive due to pain*,* usually only one quarter affected*,* in other cases*,* all quarters can be affected. Solid particles in milk. Arar– milk is ok and fine but there are wounds around the udder.“ (FGD 10).*

For ‘swelling of the udder’ (referred to as arar/barar in Somali and anjar/anjara in Oromo/Boran), it was a common notion that one form was hereditary and would reappear throughout generations. This type of swollen udder was mostly attributed to the bull, described as a “carrier male” for this condition.

*“The generation of the male one*,* all of his generation (offspring) will have swollen udder.” (FGD 8).*

In contrast, some participants would trace hereditary mastitis to the female lineage:

*“Hereditary arar (mastitis)*,* transmitted from mother to child.” (FGD 4).*

The hereditary form of ‘swollen udder’ was often associated with a specific camel type, known for high milk production, however, high milk production alone was also associated with the development of ‘swollen udder’. This was in part connected to camels producing too much milk for it to be efficiently removed and consequently the retained milk would cause the udder to swell. The relationship between the camel eco-type (hereditary), milk production and swollen udder and is illustrated by the following quote:

*“Family history*,* camels of the Horki family*,* if a Horki camel with a lot of milk gets barar (mastitis) and then give birth to another female that one will also get barar. The problem is that the calves cannot remove all the milk so the milk remaining in the udder will cause the swelling.” (FGD 10).*

The participants frequently mentioned retained milk as a cause of ‘swollen udder’. The common practice of using anti-suckling devices, broadly referred to as “teat tying” (marek in Somali, mara in the Oromo/Boran) was often highlighted as a risk factor or cause of ‘swollen udder’. Some participants explained that teat tying caused ‘swollen udder’ since it prevented the udder from being emptied:

*“The traditional way of teat tying*,* this can cause arar (mastitis)*,* because the milk is being held and it will solidify within the udder and cause inflammation.“ (FGD 3).*

For the udder problem ‘wounds on the udder/teats’, wounds were described to be caused by thorns, calves, ticks, biting insects, or the ground-dwelling naked mole-rat (*Hetereocephalus glaber*). Also, for this udder condition, participants stated that teat tying would have adverse effects on the teats including causing wounds. Despite this awareness, teat tying was widely practiced and often claimed to be necessary to ensure that there would be enough milk for the humans.

*“Mara - teat tying.* [Wounds are] *sometimes caused by the calves when they injure the teats. Sometimes as camels graze a thorn or a tree can create wounds around the udder and cause arar. When ticks accumulate around the teats*,* they can also cause this swelling.” (FGD 6).*

‘Blood in the milk’ was usually categorised as a separate udder problem, thought to be caused by the evil eye. This was mentioned in most groups in Kula Mawe (4/5) and in one group in Isiolo town (1/8):

*“Sometimes bloody milk can be caused by the evil eye*,* very common in cattle compared to camels. Evil eye can happen when somebody just passes your camel herd*,* for instances if you have camels and your neighbours don’t have the camels and he wish the herd belonged to him*,* the camel gets affected by the evil eye. Traditionally some people were known to have the evil eyes. In this area*,* you cannot find those people.*” *(FGD 5).*

However, in some groups, the idea of evil eye was rejected as something from the past, as explicitly stated in the following quote:

*“We don’t believe in that* [the evil eye] *anymore.”* (FGD 7).

### Perceptions of udder problems

**Fig. 1:**
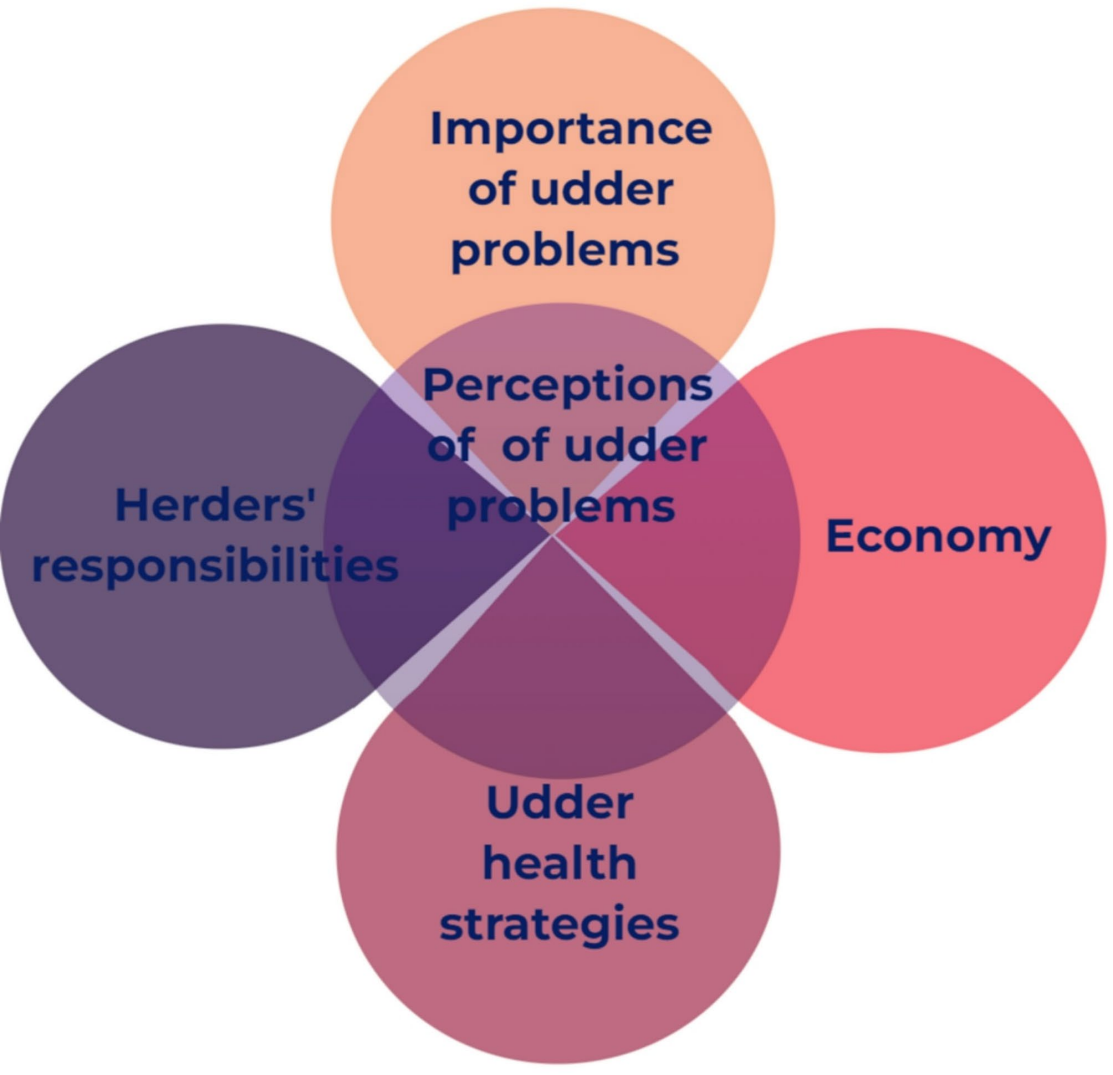
Illustration of the relationships between identified themes describing pastoralists’ perceptions of udder problems from a study with camel pastoralists in Isiolo county, Kenya

In the thematic analysis of perceptions of udder problems (data related to topics 9, 12, 14–17) codes were developed into subthemes, and the subthemes grouped into four themes: “Importance of udder problems”, “Economy”, “Herders’ responsibility” and “Udder health strategies”. Udder problems were generally regarded as multifaceted, and perceptions were affected by their perceived impact as well as available treatment and prevention options. In addition to this, we identified that treatment and prevention were largely affected by other circumstances such as logistic challenges, financial constraints and the reliability of the herders. The themes are described in the sections below, themes and subthemes are presented in Table 2. The relationships between the identified themes are illustrated in Fig. 1.

**Table 2:** Sub-themes and themes identified as describing the perceptions of udder problems in dairy camels from a study with camel pastoralists in Isiolo County, Kenya

Themes	Sub-themes
**Importance of udder problems**	• Occurrence • Minor disease • Affecting milk production • Localisation • No known treatment
**Economy**	• The value of camel milk • Reduced milk production • Negative impact of udder problems
**Herders’ responsibilities**	• Unreliable herders • Challenges for herders • Lack of knowledge
**Udder health strategies**	• Involving external help • Milking hygiene • Milk use • Culling • Change of practice

#### Importance of udder problems

Udder problems were mentioned as a common occurrence in 8 out of 13 groups. Several groups mentioned that it was more common in lactating camels with a high milk production.

*“When camels are giving birth and lactating*,* it’s the most common disease in camels.” (FGD 10).*

Despite udder problems being described as occurring regularly, it was only brought forward as a prioritised disease in one group. The effect of udder problems was perceived to be locally restricted to only the udder itself and the condition was generally not associated with high mortality. These factors were part of the rationale for not considering udder problems a highly prioritised disease.

*“Arar (mastitis) and qarfat (contagious skin necrosis) are there but they cannot kill the animal so qandich (haemorrhagic septicemia) is more important.” (FGD 6).*

However, in some groups, udder problems were given larger importance. In these groups characteristics such as impaired milk production, the risk of permanent loss of milk production for the affected quarters, the negative impact on calf health and the lack of effective treatment seemed to contribute to perception of higher priority.

*“[Mastitis is…] important because we are dependent on milk*,* and arar (mastitis) destroys the udder*.” (FGD 2).

Low milk production was mentioned as an important sign for several other diseases, in almost all groups, and this was considered an important factor when prioritising diseases:

“*If a camel is not healthy*,* you can see changes in behaviour*,* low milk production*,* not enough milk for the calf*,* rough body condition*,* when sick it does not produce milk.” (FGD 7).*

Generally, mastitis was perceived as more important among people that were involved in milk trade and seemed to be regarded as less important among camel owners.

#### Economy

Milk production was described by the participants as essential for their livelihoods and decreased milk production as having negative effects on the people depending on the milk as well as negative impact on calf health. Production losses could affect humans negatively in the form of economic losses (reduced household income) and less milk available for household consumption.

*“Camel milk is a very important commodity for us. It helps the herder*,* from the market to the household*,* it is important to a lot of people.” (FGD 8).*

The risk of negative effects for the calf, such as reduced growth, disease and potentially increased calf mortality was also raised.

The impact of udder health problems was discussed regarding the individual camel and for the camel owner. For camel owners that were selling their milk, udder problems were considered a risk factor for having the milk rejected by the milk cooperative leading to reduced income, and ultimately if the disease progressed, to having to sell or slaughter the animal and thus suffer from a reduced herd size.

*“Sometimes when we are sending the milk*,* we receive information that the milk has been rejected and been spoiled and this is a lot to us.” (FGD 2).*

For the individual camel, impact mentioned were issues of recurring infections in the same camel, loss of function in the affected quarter and sometimes loss of the entire udder.

*“*[Mastitis is] *Very common*,* I have three cases in my herd. They have had it for a long time. It usually affects when the camel is lactating and comes back every new lactation. Mainly common in high milk producing camels.” (FGD 8).*

Permanent loss of milk producing function and milking ability in the affected udder quarter was frequently mentioned and this appeared to be a common disease outcome (mentioned in ten groups). The loss of function was sometimes described as affecting only one quarter but sometimes expanded to include the whole udder and sometimes leading to an actual shedding of the infected tissue:

*“Sometimes the whole quarter just drops of*,* sometimes the whole udder.” (FGD 2).*

Participants largely agreed on that once the camel completely lost its ability to produce milk, it would be sold for slaughter since it no longer could generate income. Selling it would also partly compensate for the production losses. However, some participants stated that camels that were being sold would not necessarily be replaced and that the decision depended on the owner.

*“If the whole udder is affected*,* we just sell them. It has no importance because it cannot produce milk. Even if the animal contracts arar frequently we will just keep it until all the teats are gone*,* then we sell it.” (FGD 6).*

#### Herders’ responsibilities

Participants often emphasised the important role in employing good herders as a cornerstone in maintaining healthy animals, also in relation to udder health. Improving the knowledge of the herders by providing education and proper instructions were seen as ways to improve the herd health.

*“It depends a lot on the herder if he can be very keen and do much work*,* that will reduce arar [udder problems].” (FGD 3).*

In contrast, a heavy burden of responsibility was placed on the herders and camel owners often discussed herders as a factor that was difficult to influence. The tasks that herders could fail to comply with ranged from removing teat tying, maintaining good hygiene during milking, adhering to a milking order, finding good pasture for the animals, respecting withdrawal times for milk, detecting, observing and treating disease in time. In addition, camel owners would often agree that herders were lazy, cared more about receiving a salary than the health of the camels, lacked necessary experience and would seldom follow instructions.

The employment of herders was seen as a relatively recent development and participants acknowledged that this major change in management could affect the well-being of the camels. Finding herders with enough experience of camel management and milk production was mentioned as a problem.

*“In old times people lived in the bush*,* they stayed with the animals*,* now you just hire someone that doesn’t even know nothing about camels*,* as an owner and a herder you don’t know what your camels are feeding on” (FGD 12).*

Parallel with the prevalent negative attitudes towards herders, participants acknowledged that camel herding was labour-intensive and that herders would face many challenges.

*“It’s a very difficult task*,* managing the camels.” (FGD 7).*

In summary, employed herders were seen as a fundamental pre-requisite but also a possible major obstacle to good animal health.

#### Udder health strategies

A number of different approaches to handle or prevent udder problems, including traditional remedies, antibiotic treatment and milking strategies, were discussed by the participants. These approaches were largely based on the perceived cause of the udder problem. Mostly, similar practices were applied to deal with camels suffering from udder disease. In three groups, some participants stated that udder problems did not need to be treated at all but would automatically heal. Other approaches included to separate the camel from the herd and milk camels with udder problems last, abstain from milking the affected quarter and leave it for the calf. This was either motivated by difficulties in milking the inflamed quarter due to the camel being in pain, and/or to avoid mixing the infected milk with the healthy milk.

*“Painful to touch the udder but they [the camels] often allow the calf to suckle. If we find these signs*,* we leave it to the calf to suckle. In case of some quarters affected and some healthy you leave the affected to the baby and milk the others. You milk the healthy ones and leave the other ones for the baby.” (FGD 6).*

In contrast, in three of the FGDs, removing mastitic milk from the affected quarter was stated as an important strategy to turn the condition around.

*“In case of swelling you just milk all the milk from the udder and remove everything until the swelling is reduced. “ (FGD 10).*

During the analysis, it became apparent that treatment of mastitis did not follow any particular guidelines but differed depending on the perceived cause and previous experience. All groups would use antibiotics to treat udder problems. Oxytetracycline (Adamycine) or a combination of penicillin and streptomycine (PenStrep), usually injected intramuscularly, were mentioned in all groups as a regular treatment of udder health problems. Drug dosage, duration of treatment or preferred type of antibiotic varied between and within groups as well as with the individual case. Routes of administration for different medicines also varied, from injection (subcutaneous, intravenous, intramuscular) to intramammary or topical treatment. The phenomenon of “blocked teats” due to udder problems was often discussed, including various solutions.

*“Treat with tetracyclines*,* but when the teat is blocked*,* they use the tubes (penicillin) to open up. Inject through the hole of the teat and remove the blockage.” (FGD 12).*

Some groups would use traditional methods, such as herbal preparations, sometimes fed orally to the camel and sometimes used topically on the udder.

*“Traditionally we would treat it with a type of herb and apply around the udder and it will heal it.” (FGD 11).*

Using the fat from a sheep was advocated as medicine by some pastoralists, particularly to “open up” blocked teats. Cauterisation (branding with hot iron) is a common treatment for different types of conditions among camel pastoralist and was also often brought up as a treatment option.

*“Some people brand with metal if the swelling goes up to the stomach. Other people use branding around the udder.” (FGD 11).*

In addition to somatic treatments, spiritual/magical strategies, such as prayer or organising a religious ceremony, were of particular importance when it came to removing the evil eye from a camel and mentioned in two groups. Traditionally, when it was suspected that someone had given the evil eye to a camel, the owner would ask that person to remove it from the camel. However, the participants expressed that this was no longer commonly practiced, due to fear of offending the person suspected of the evil eye.

*“This evil eye is generally caused because of jealousy. Traditionally it used to be associated with a special tribe but nowadays it is caused by jealousy*,* someone who doesn’t have a camel and they want yours. Sometimes evil eye affects the male so it can no longer mount.*

*Male participant 1: This evil eye is in every tribe*,* it’s everywhere.*

*Male participant 4: Sometimes people suspected of having evil eye can be moving around your herd and then they advise them to stay away from your herd. That is how it was done traditionally but these days there is that fear*,* they can’t even tell them to stay away.“ (FGD 6).*

There was consensus in and between groups that milk from unhealthy udders should be separated from other milk, or all milk would be spoiled. This was discussed as being of particular relevance, since spoiled milk would be rejected at the market and result in loss of income. Regarding the use of milk from mastitic udders, conflicting statements were often made. Although some participants were of the opinion that mastitic milk should be left for the calf or milked to the ground, others stated that it should not be used at all. Some mentioned that the decision rested on the herders.

*“Do not mix with milk from healthy camels. Some leave it for the calf. It depends on the herder*,* but they do not mix it with the milk that’s going to market.” (FGD 10).*

Some practices that could be considered as preventative udder health measures were mentioned to be implemented in the camel herds represented in the groups, see Table 3.

**Table 3:** Strategies applied to prevent or minimise udder problems in camel herds, mentioned in focus group discussions with camel pastoralists in Isiolo County, Kenya, in 2022

Udder health strategies	Number of groups mentioned in (*n*)
Cull chronic cases	8
Improve the milking hygiene	8
Milk the affected one last	6
Remove colostrum or milk	3
Stop teat tying	3
Acaricides	2
Cull carrier camel	2
Isolate the affected camel	2
Education of herders	1

Milking the camels in a defined order to minimise spread of disease (using milking order) in the herd was mentioned in 6/13 groups and this was a strategy well-recognised by participants and often employed. The following quote illustrates an approach often used by the participants:

*“Milking the old and the ones with disease last.“ (FGD 10).*

The use of acaricides to protect animals from tick infestation was described as a way to prevent udder problems due to bites and mentioned in 2/13 groups.

*“Spray animals to protect them from ticks that can cause udder problems.” (FGD 1).*

Although teat tying was commonly associated with udder problems, the practice was regarded as a fundamental measure to prevent milk from being drunk by the calf. However, some modifications to reduce its harmful impact were described as a preventive measure in 3/13 groups:

*“These days we do not use the teat tying like we used to do because we believe it increases the swelling. We advise the herders that even if they tie*,* they should tie very carefully/loosely. Even if the herders tie*,* they should rotate so they don’t tie the same quarters every day.” (FGD 6).*

In the case of hereditary ‘swollen udder’, believed to be caused by carrier males, using a different bull to reduce the spread was described as a preventive measure in two groups:

*“Remove the bull from the herd. It would have been better to control it by culling it and getting rid of the transmitting bull and remove all this*,* but it is not possible.” (FGD 4).*

In some groups, parallel with airing beliefs about ‘swollen udder’ being a hereditary condition, participants would discuss improved milking hygiene as a preventive measure against udder disease. Unclean milking practices, such as not washing hands in connection with milking, using dirty materials for teat tying, applying dung on the teats to prevent the calf from suckling or milking healthy camels after milking camels with mastitis were considered risk factors that could be avoided. Adhering to hygienic practices during milking was mentioned as a way to control udder problems, however lack of water was considered a major constraint.

*“When you milk the camel with udder problems*,* you will not go the next one until you have washed your hands. This is because otherwise udder problems are transmitted to the healthy ones*,* they will become affected unless you wash your hands. When you are not washing your hands*,* you may affect all camels in your herds. You have some people where all their milking camels are affected by swollen udders*,* and this is because they did not wash their hands and it spread. “ (*FGD 12).

The issue of transmission often yielded many contradictory answers in the same groups and many elaborations on contagiousness were offered; in this aspect, causes of the udder problem were claimed to be the hands of the milker, the milk or the milking bucket itself:

*“They cannot milk into the same container as healthy camels*,* it is transmissible from the bucket*,* the milk from the affected camel can infect the healthy one.[…] It is not contagious through body contact*,* only by infected milk.” (*FGD 4).

### Perceptions of measures for preventing udder problems

In the thematic analysis of perceptions of preventive measures (data related to topics 18–22), codes were developed and grouped into four emerging themes: “Feasibility”, “Accessibility”, “Sustainability” and “Effectiveness” together with identified sub-themes (see Table 4). The emerging themes were positively connected with the adoption and implementation of the suggested interventions and on the contrary, the inversion of the themes, such as something being regarded as “unfeasible” or “unsustainable”, was considered major hindrances.

**Table 4:** Identified themes and sub-themes for interventions promoting udder health as discussed in focus group discussions with camel milk value chain stakeholders in Isiolo County, Kenya, 2022

Emerging themes	Sub-themes
**Feasibility**	• Disruption of milking process • Herder compliance • Labour-intensity • Market demand
**Accessibility**	• Access to equipment • Access to knowledge • Availability of material
**Effectiveness**	• Visualisation of mastitis • Detectable results
**Sustainability**	• Affordability • Re-stocking of material • Safe to use for the animals

#### Feasibility

Milking camels under traditional pastoral management was described as a sensitive and labour-intensive process, where any kind of disturbances before or during the milking could interfere with the milk let-down. Control measures that were positively received were those that were perceived to have minimal interference with the regular milking routine, such as application of hand disinfectant between camels or teat dipping. Control measures that could interfere with milk let-down were generally deemed as unfeasible. Measures regarded as unfeasible included the use of gloves that were to be changed between camels and the use of wet tissues to wipe the teats.

*“[Discussing teat dipping] After milking is very possible. If before*,* the camel will hold the milk.“ (FGD 1).*

The unpredictable nature of the individual camel was often mentioned as something that would influence the feasibility of some interventions. Participants would state that some camels would be more accepting whereas others more easily could become aggressive, thus posing a risk to the herders and disturbing the routine. This was not necessarily seen as a hurdle but rather highlighting this as a factor that was beyond human control.

*“[Camels are] very sensitive. They cannot do that. Camels are aggressive*,* they understand what you are doing*,* and they can kick you. Camel allows you to milk but will not allow you do that. Maybe you can restrain the camel. But if you restrain them*,* they will withhold the milk. It is also labour intensive. The herder cannot do all that work. Camels only allow them to milk them peacefully but when you dip them*,* they become aggressive. Cows accept such things but not camels.” (FGD 10).*

Reliability of the herders was often considered something that would impact feasibility and that was associated with difficulties in implementing any interventions. The participants would often state that the attitudes and knowledge of the herders were critical, but that herders were difficult to control, lacked sufficient knowledge, were unreliable or overworked.

*“Tricky because herders are ignorant. You may give hand disinfectant for them to use*,* and they may fail to use it and only use it when the owner is around. Herders are very ignorant.” (FGD 9).*

In two groups, participants described that the milk-cooperatives were in a position of power and could put pressure on milk producers to comply with their requirements. In such a scenario, the milk producers would have to adopt the suggested strategies, irrespective of potential hindrances in order to be able to deliver milk.

*“In case there are strict rules saying that we have to use it to milk our camels*,* for instance if the cooperative demands it*,* we will use it.” (FGD 6).*

#### Accessibility

This theme encompassed both access to equipment and/or expendables and the access to knowledge of how to use the suggested interventions. The remoteness of most camel dairy herds in addition to the migratory nature of camel pastoralism makes access to equipment imperative. Accessibility to equipment referred to both spatial distance as well as availability of material to purchase.

*“[Discussing using hand disinfectant] Could have been good*,* problem is that you can’t find it in the bush. If you can access it*,* you can use it.“ (FGD 12).*

Furthermore, participants often reiterated the importance of knowledge and their wish to acquire more information on how to improve the health of their camels and how to use some of the suggested interventions.

*“[I have] heard about the CMT (California Mastitis Test) and seen a demonstration*,* this has been taken to our boma. If we can get it*,* we could use it*,* if we can get the knowledge and access.” (FGD 4).*

#### Sustainability

Interventions depending on consistent restocking of expendables, such as the use of gloves or tissues, were largely dismissed. These measures were perceived negatively partly because of the added costs. Pastoral camel milk production is a low-input system, and economic margins can be narrow. Furthermore, it was discussed that some equipment was disposable and could not be re-used, making the use unsustainable.

*“It is available but expensive if you need to change after every camel.” (FGD 1).*

Participants often voiced concerns about interventions, such as hand disinfectant or teat dip, being harmful to the camels or the suckling calves. Safety for both camels and humans had to be guaranteed for the participants to consider an intervention.

#### Effectiveness

The possibility of achieving detectable results seemed to be a fundamental and understandable motivator, shaping the attitudes of the participants towards the suggested interventions. Given the limited monitoring, for example individual milk yield and milk quality measures in place in pastoralist camel milk production, the effectiveness of udder health promoting interventions is difficult to assure. As such, the positive effects of some interventions were questioned by some participants.

*“We have milked the sick one [camel] first and then milked the others*,* but I have not seen any spread [of infection]*,* so this is not true. If this thing is really transmitted through dirty hands*,* if you have ten herds and we milk the affected ones first and then the healthy ones*,* how come this is not spreading?” (FGD 6).*

Mirroring that, the California Mastitis Test, an animal side test which is based on the subjective assessments of increasing viscosity of the milk after being mixed with the test reagent, was viewed as a very useful tool, especially among participants who had experience from using it.

*“We were shown a CMT-test to distinguish between healthy and unhealthy milk from the camel. It was shown very clearly” (FGD 3).*

The opinions captured in the FGDs regarding suggested udder health interventions have been summarised in Table 5.

**Table 5:** Distribution of positive versus negative views with regards to the applicability of udder health interventions mentioned in 13 focus group discussions (FGDs) with camel pastoralists in Isiolo County, Kenya, 2022. The table shows the number of positive/negative viewpoints that were voiced per intervention in total, not using each FGD as the point of measure. Direct quotes exemplifying some of the voiced opinions is included for each intervention

Suggested intervention	Positive	Negative	Positive comment	Negative comment
California Mastitis Test	12	2	“We have tested using the CMT and could clearly see the diseased ones and the healthy ones.”	“It can be difficult to use because the herders are with the camels and even if they are given instructions” they may do or not, you cannot control
Milking order	12	3	“[We are] already doing that, milking the old and the ones with disease last.”	“This is not possible because you can’t control the baby that is coming from the boma (that is what is deciding the milking order).”
Teat dipping	12	5	“After milking is very possible. If before, the camel will hold the milk.”	“Not possible to use teat dipping, camel will not allow it. They could kick the bucket of milk so it would spill.”
Hand disinfectant	8	8	“This could be used, problem is that people use water but sometimes it is not available so if they could have an alternative that could be used.”	“[We] don’t us it. It is not possible. It can cause harm to the udder itself. Some disinfectants have spirit inside them, they can cause harm to the herd itself.”
Wiping udder with cloth	5	9	“It depends on the camel, some would only allow the calf to suckle, some you can help and for them, wet wipes would be very possible.”	“Impossible, camels will not allow wet things touching their teats. Herders usually clean with their bare hands without water. Before milking they wash their hands.”
Using gloves	5	12	“[We] have never tried using gloves but if they could get them maybe they could use them. The problem is that you have to find them and buy them. Camels are far away in the bush, they cannot access the gloves. Maybe the camel owner is in Isiolo and the herder is somewhere far away and when the gloves are used up, he cannot get new gloves.”	“Using gloves is not possible, the teats are usually hard in comparison with the cattle ones, use of gloves is not easy. Gloves slide.”

## Discussion

The way the participants in this study discussed mastitis reflect the contemporary scientific understanding of mastitis as a complex and multifactorial disease. Moreover, the results pertaining to the clinical signs of mastitis demonstrated the vast knowledge that pastoralists possess regarding how diseases they see in their animals present. The close relationship with, and dependency on, their livestock have shaped the skills to detect clinical disease [32] and most participants could list a wide range of signs associated with clinical mastitis. In contrast, SCM was not mentioned in any of the discussions. This is in line with previous studies highlighting the difficulties for pastoralists to detect subclinical mastitis relying solely on sensory inputs [3, 7]. As subclinical mastitis has proven to be much more prevalent than clinical mastitis in similar contexts [3], this finding stresses the importance of interventions to improve udder health in pastoralist camels. Participants offered multiple explanatory models for the development of mastitis. The models frequently included a mix between elements that in contemporary mastitis science would be classified as “causes of mastitis” (i.e. an ascending intramammary infection of bacterial origin) and “risk factors for mastitis”. This way of developing explanatory models built on local knowledge, observations and practice has been reported also in other pastoralists communities [33]. Some participants challenged the notion of indirect transmission of mastitis, referring to their own experiences of milking sick animals before healthy ones without spreading clinical disease. Multiple views were expressed, listing infected milk as a source of infection that could transmit mastitis to healthy milk if the milk was mixed, and the milk vessel itself being regarded as the source of infection by some participants. Participants’ observed association between high milk production and development of mastitis is interesting, as high milk production has been associated with mastitis in camels [34] and also in cattle [35]. Udder health problems caused by trauma to the udder skin were identified as a separate form of mastitis by a majority of the participants. Skin lesions have previously been associated with an increased risk of mastitis in camels [3, 36], as have ticks. In a study with Borana pastoralists in Ethiopia, ticks were perceived to be a major burden for cattle and the udder was identified as a predilection site, with tick infestation perceived to cause blinding of teats and have a negative impact on milk production [37]. In the harsh study setting, skin trauma could be a noteworthy cause of mastitis that needs to be addressed in future prevention strategies. The commonly mentioned association between family lineage and mastitis could be an explanation to a number of prevailing conditions. First of all, in cattle, sensitivity to mastitis is in part linked to genetics and has been introduced as a selective breeding criterion [38]. The notion that mastitis could be transmitted from mother to calf could also illustrate the phenomenon of calves becoming carriers of contagious mastitis pathogens through the consumption of infected milk, increasing the risk of intramammary infection later in life. This has been shown for *Staphylococcus (S.) aureus* in dairy cattle [39, 40]. Furthermore, it has been shown that camel calves can become carriers of *Streptococcus (Str.) agalactiae* through transmission from their mothers [41].

According to the results, camel pastoralists in Isiolo seem to operate in the intersection between modern veterinary knowledge and deeply rooted traditions and cultural beliefs. This was illustrated in the mentions of evil eye causing blood in the milk, and regarding the practice of teat tying. The concept of the evil eye is often associated with jealousy, and it is believed that only certain people possess the evil eye [12]. In accordance with the perceptions of several other pastoralist groups in the region [7, 12, 13], the evil eye was attributed specifically to clinical mastitis with blood in the milk. For cases of clinical mastitis, the appearance of the milk can change drastically, with the milk containing flakes or clots, becoming thicker or more watery than normal and sometimes stained or mixed with blood. However, there is no correlation between causative agent and the clinical presentation with regards to the milk. Many participants explained that the cultural attitudes surrounding the belief of evil eye as a cause of udder problems were changing, making it more complicated to seek treatment according to their traditions. Similar views were voiced in a study by Amenu et al. [12] investigating perceptions about (bovine) udder health in Borana pastoralists, where it was suggested that this development could be positive in shifting traditional/magical treatment for the evil eye to the use of antibiotics which could have positive implications for animal welfare and bacteriological cure. Previous research point to the importance of participatory involvement of actors such as milk collection centres and livestock keepers in such processes to co-construct and adapt interventions to pastoralist settings and the local context [42]. Regarding the traditional practice of teat tying, many participants agreed that it was a harmful practice and a risk factor for udder problems causing retained milk and skin damage to the teats, but that they despite this knowledge would continue the practice. Traditionally, camel milk was only used for household consumption and selling it was regarded as something that would bring bad luck. Giving another example of the changing environment in which the studied pastoralists operate, this custom changed a few decades ago. What initially started with unorganised female vendors hawking surplus milk to individual households has now developed into a more intensified production, with a value chain involving a wide range of stakeholders and women owning camels. The commodification of camel milk has been viewed partly as a response to the increasing sedentarisation of nomadic pastoralists, and in part to their subsequent adaptation strategies, such as livestock diversification [1]. The recent changes in the camel pastoralist lifestyle, such as women now owning camels, owners no longer staying with the herds, employment of hired labourers and monetary values replacing traditional ones, are happening parallel to other major changes, such as climate change and development of critical infrastructure in pastoralist rangeland.

Many participating camel owners explicitly stated that camel milk was essential to their livelihoods, but nevertheless, little importance was given to mastitis in comparison with other diseases. This is similar to what has been reported from mixed livestock pastoralists in Ethiopia [43]. Low milk production is a common consequence of mastitis, but this was not listed as a clinical sign for mastitis specifically. In contrast, decreased milk production and its negative consequences on household income, calf nutrition and food security were frequently mentioned when describing many other diseases. Given that camels under pastoralist management produce moderate quantities of milk and the individual yield is usually not recorded, variations in milk yield are likely difficult to detect for the owners, unless very large. The variation in milk yield between the dry and the rainy season, on the other hand, differs significantly [44] and can be reduced by 60% during the dry season (personal communication, Tawakal ltd). As all animals are affected by the dry season at the same time, its negative impact on milk production will be tangible and easily detectable, especially at herd level. The results show that camel pastoralists face many challenges, of which infectious diseases constitute a constant threat. The fact that in most cases mastitis is not fatal, it is restricted to the udder and often confined to one quarter, as well as that most pastoralists are aware of treatment options probably served as a rationale for not ranking mastitis as an important disease. In comparison to highly contagious diseases with high mortality and limited treatment options, such as anthrax or camel pox, mastitis was regarded as a less important disease. This corroborates previous knowledge from other pastoralist groups where diseases with direct effects on the economy, cultural values and utilisation of ecosystem services are the main factors determining the importance [32]. Diseases that affect many animals at once with a high mortality are generally ranked as more important among pastoralist groups [24, 45].

Udder health problems were perceived to negatively impact household income through two main routes: by impairing the function of the mammary gland until milk production ceased, and by deteriorating the quality of milk to the extent that it would be at risk of being rejected by the bulking centre (usually a milk cooperative). Among the participants it was common practice to keep camels in the herd until no functional udder quarters remained. Loss of secretary function in the mammary gland is usually the end stage of a chronic intramammary infection, most often caused by contagious pathogens, such as *S. aureus* or *Str. agalactiae*. Keeping chronic carriers in the herd poses a major risk of transmission to other animals. In high-income countries, the practice of culling chronic carriers has been recommended since the 1960’s [46], and proven successful in eradicating or reducing contagious udder pathogens and improving udder health. If implemented, the practice would probably have the same positive impact on udder herd health in camel populations. In the Horn of Africa, however, the price of camels by far exceeds the price of all other livestock, with lactating females being particularly valuable [47]. In pastoral populations, the high economic and cultural value of camels in combination with high levels of food insecurity and poverty will thus make this recommendation inappropriate and not likely to be implemented. The frequently mentioned fear of having milk rejected from bulking centres illustrates the important role the milk cooperatives represent in the camel milk value chain. Efforts aiming to improve milking practices and promoting a defined milk quality standard that could serve as an incentive for improving camel udder health should therefore target both the primary producers/pastoralists and the bulking centres/milk cooperatives. Educational efforts towards women, who are often in charge of milk handling and vending, have shown to partly be efficient in improving attitudes and practices regarding milk hygiene [48]. Penalties or premiums connected to milk quality is common in dairy industry worldwide and has been used as tool for improving udder health. In a study on motivations for improving udder health in dairy farms in the Netherlands, it was shown that equal importance was given to pride in healthy animals and factors affecting the economy. It was also concluded that financial penalties were perceived as being a more efficient motivator, than being paid premium prices for better quality milk [49].

It has been concluded that compliance and success of disease control programs is dependent on the priorities of the program matching the priorities of the livestock keepers [43]. To ensure compliance by animal owners, the advice given needs to be understandable and follow the principles as formulated by Dodd [46]: “To be accepted, a control must cost much less than the losses caused by the disease, it must be relatively simple to carry out, there should be good experimental evidence that the control works under a range of conditions, and it must be obvious to the farmers who adopt the method that [clinical] mastitis is much reduced.” The results confirm that the advice offered to participants (by cooperatives, research institutes or NGOs) on acute mastitis can be confusing as they are based on concepts that are not fully known. understood, or relevant in the participants local reality. As an example, many of the participants struggled to see the association between their own experience (mastitis not being transmitted) and advice that is mainly focused on improved milking hygiene, meant to prevent transmission of SCM. When conducting a study on camel calf health in northern Kenya, Kaufmann [50] concluded that knowledge of the disease perceptions among the target group is crucial when designing and delivering animal health advice so that animal owners can understand and act on it, and thus ensure implementation of the interventions.

Udder health strategies mentioned by the participants could be divided in preventative measures and treatment of clinical cases. As has been reported previously, there was large inconsistency in treatment strategies for udder problems [12]. The use of syringes or tubes to “open up” blocked teats was worrying since the teat anatomy in camels is very different from other ruminant dairy species. In camels, there are 2–3 long and narrow teat canals per teat [51], thus making them susceptible to trauma if products developed for cattle or small ruminants are being used. Furthermore, when using intramammary infusions, special care needs to be taken to ensure hygienic conditions and to avoid introducing new pathogens into the mammary gland. Without adequate training, there is a major risk of causing damage [52]. The use of injectable antibiotics was common practice to treat udder health problems, and oxytetracycline and/or a combination of penicillin and streptomycin were largely used, in line with previous findings [3]. The use of antibiotics is often a necessary step to ensure bacterial cure for intramammary infections, however the drug needs to be selected according to substance characteristics making it suitable for treatment of udder infections, the pathogen in question and its susceptibility to the selected substance. Oxytetracycline might not be the optimal drug of choice as resistance is widespread in many bacterial species involved in intramammary infections in the region [3, 41, 53, 54], the substance has been shown to have a reduced antibacterial activity in mastitic milk from cows [55, 56] and a poor efficacy in the bovine udder [57, 58].

Milking animals in a certain order, taking in to account the udder health status of each individual to hamper the spread of contagious udder pathogens, is a simple and efficient method to improve the herd udder health [59]. This strategy was commonly implemented in many of the participants’ herds and provides an important stepping stone towards improved udder health. The current strategy was often to milk cases of clinical mastitis last, which is a promising approach, however, it completely omits SCM-cases that could still serve as reservoirs for contagious transmission. In addition, it has been shown that the risk of mastitis increases with the age of the camel and the stage of lactation [3]. If more factors were to be included, such as CMT-results, age and stage of lactation behind the rationale for the milking orders that are currently in place, this could be a very good starting point for a more long-term udder health strategy.

The use of employed herdsmen has been listed as the main economic investment in pastoralist camel milk production [60] and thus, heavy responsibilities are placed on the herders. In this study, the herders were viewed as key players for keeping the camels healthy, safe from thieves and predators, and delivering milk regularly. The fact that camel herding is labour-intensive was widely acknowledged by the participants, yet, herders were consistently viewed as a major constraint to a successful business. In a study on awareness of milk hygiene in camel herds, herdsmen had significantly less knowledge about hygienic practices during milking and handling of camel milk compared with female milk vendors and members of milk cooperatives [61]. In areas where the herdsmen had improved knowledge, the prevailing conditions that the herds were operating under still obstructed optimal practices. Furthermore, when comparing the total viable (bacterial) count (TVC), number of coliforms and *S. aureus* in swabs taken from camel udders and milkers’ hands, the overall result indicated that the bacterial load on the milkers’ hands was higher than on the udder [4]. The lack of knowledge on adequate hygiene practices among employed herdsmen poses a risk, both to the udder health of the camels and for introducing contamination to the milk already at herd level [62]. It has been shown that lack of support from employers in terms of education and raising awareness is a challenge for farm workers [63] and there is also an association between perceived stress levels and health problems in farm workers and a high disease burden in dairy herds [64]. Including herders is therefore imperative for improving the udder health, overall milk hygiene and relieve some of the stress on the employed herdsmen. A limitation to the present study was the lack of herder participation in the FGDs as only 12 out of 85 participants were employed herders. A number of factors negatively influenced the possibility for herders to attend the FGDs; the herders are employed and therefore would struggle to get “time off” to attend an FGD, the sheer remoteness of the camel herd makes transport options difficult as well as transport costs, and finding a replacement herder for the time the other one is absent is another challenge. Another limitation in including the attending herders’ perspectives was that the authors were repeatedly advised not to separate herders and camel owners in the FGDs since this could create suspicion among the camel owners. However, by using mixed groups there was a fundamental inequality in the power distribution between herders (employees) and camel owners (employers). Camel owners would freely voice negative attitudes towards herders in the presence of the same. This power imbalance is likely to have inhibited herders from freely expressing their opinions and concerns to maintain a respectful position towards the employer category.

The attitudes to the suggested preventive measures were multifaceted, but some distinct trends were still identified in the thematic analysis. The discussion about the measures largely revolved around accessibility, availability and feasibility. Aids that result in a very distinct and visible result, such as the CMT, were clearly preferred over control measures that required a lot of disposable material and increased labour, such as the use of plastic gloves that are changed between every milking or wiping the teats with wet tissues prior to milking. It has been shown that implementation of improved milking hygiene measures can be obstructed by intrinsic factors, such as personal habits, not perceiving udder health as a priority on the farm and physical barriers, such as time, money and labour [65]. The participants had mostly positive attitudes towards the use of a milking order, as this was often partly already implemented in the existing routine, was not perceived to interfere with milk let down, did not involve any extra equipment and thus was feasible, affordable, accessible and sustainable. The findings that the use of CMT, hand disinfectant and milking order could potentially be accepted for use in pastoralist herds are very promising and could form the basis for a pastoralist camel udder health strategy that now needs to be piloted and evaluated.

## Conclusion

Camel pastoralists in Isiolo are skilled at detecting clinical mastitis and have developed a number of treatment and prevention strategies to maintain a good udder health. Furthermore, they have good awareness of milking hygiene and what practices to avoid but resort to local explanatory models and causal relationships when their knowledge is incomplete. However, with the camel milk production industry rapidly undergoing extensive changes, modern concepts and deeply rooted traditions are often adopted in parallel, sometimes resulting in conflicting views on best practices. Despite camel milk being the most important commodity to camel pastoralists, the detrimental effects of mastitis, both clinical and subclinical, on milk production were poorly understood across all stakeholder categories that were included in the study. The use of hand disinfectant, CMT and a milking order were positively perceived by the participants. Another important finding was the crucial role that employed herdsmen play in keeping the camels healthy and ensuring good milk hygiene. Educational efforts targeting herders, together with selected interventions and strategic milk hygiene work driven by economic incentives could form the basis for a future control strategy that should be tested and evaluated in pastoralist camel herds and the camel milk value chain.

## Electronic supplementary material

Below is the link to the electronic supplementary material.

Supplementary material 1: Topic guide

## Data Availability

Data are available from the SVA Institutional Data Access (contact via corresponding authour) for researchers who meet the criteria for access.

## References

[1] Anderson DM, Elliott H, Kochore HH, Lochery E. Camel herders, middlewomen, and urban milk bars: the commodification of camel milk in Kenya. J East Afr Stud [Internet]. 2012 Aug 1 [cited 2021 Jan 18];6(3):383–404. Available from: 10.1080/17531055.2012.696886

[2] Noor IM, Guliye AY, Tariq M, Bebe BO. Assessment of camel and camel milk marketing practices in an emerging peri-urban production system in Isiolo County, Kenya. Pastor Res Policy Pract [Internet]. 2013 Dec 2 [cited 2021 Jan 18];3(1):28. Available from: 10.1186/2041-7136-3-28

[3] Seligsohn D, Nyman AK, Younan M, Sake W, Persson Y, Bornstein S et al. Subclinical mastitis in pastoralist dairy camel herds in Isiolo, Kenya: Prevalence, risk factors, and antimicrobial susceptibility. J Dairy Sci [Internet]. 2020 May 1 [cited 2021 Feb 23];103(5):4717–31. Available from: https://www.sciencedirect.com/science/article/pii/S0022030220302010 10.3168/jds.2019-17701 32171518

[4] Odongo NO, Lamuka PO, Matofari JW, Abong GO. Risk factors associated with the post-harvest loss of milk along camel milk value chain in Isiolo County, Kenya. Afr J Agric Res [Internet]. 2016 Feb 25 [cited 2021 Jan 18];11(8):674–82. Available from: https://academicjournals.org/journal/AJAR/article-abstract/3717A0E57314

[5] Toroitich CK. Prevalence Of Mastitis And Associated Risk Factors In Lactating One-Humped Camels In West Pokot County, Kenya [Internet] [MSc thesis]. [Nairobi, Kenya]: University of Nairobi; 2013 [cited 2020 Dec 10]. Available from: http://erepository.uonbi.ac.ke/handle/11295/62291

[6] Abbas B, Al-Qarawi AA, Al-Hawas A. The ethnoveterinary knowledge and practice of traditional healers in Qassim Region, Saudi Arabia. J Arid Environ [Internet]. 2002 Mar 1 [cited 2021 Mar 13];50(3):367–79. Available from: https://www.sciencedirect.com/science/article/pii/S0140196301909048

[7] Abera M, Abdi O, Abunna F, Megersa B. Udder health problems and major bacterial causes of camel mastitis in Jijiga, Eastern Ethiopia: implication for impacting food security. Trop Anim Health Prod [Internet]. 2010 Mar 1 [cited 2020 Dec 10];42(3):341–7. Available from: 10.1007/s11250-009-9424-6 10.1007/s11250-009-9424-6 19731066

[8] Raziq A, de Verdier K, Younas M. Ethnoveterinary treatments by dromedary camel herders in the Suleiman Mountainous Region in Pakistan: an observation and questionnaire study. J Ethnobiol Ethnomedicine [Internet]. 2010 Jun 21 [cited 2021 Mar 13];6(16). Available from: 10.1186/1746-4269-6-16 10.1186/1746-4269-6-16 PMC3224957 20565919

[9] Tuteja FC, Dixit SK, Kumar S, Patil NV, Singh JP. Traditional treatment practices against camel diseases in Rajasthan. J Camel Pract Res [Internet]. 2011 [cited 2021 Mar 13];18(2):231–42. Available from: https://www.indianjournals.com/ijor.aspx?target=ijor:jcpr%26;volume=18%26;issue=2%26;article=012

[10] Farah Z, Mollet M, Younan M, Dahir R. Camel dairy in Somalia: Limiting factors and development potential. Livest Sci [Internet]. 2007 Jun 1 [cited 2020 Dec 10];110(1):187–91. Available from: http://www.sciencedirect.com/science/article/pii/S187114130600463X

[11] Chengat Prakashbabu B, Cardwell JM, Craighead L, Ndour APN, Yempabou D, Ba E et al. We never boil our milk, it will cause sore udders and mastitis in our cows- consumption practices, knowledge and milk safety awareness in Senegal. BMC Public Health [Internet]. 2020 Dec [cited 2021 Mar 12];20(1):742. Available from: https://bmcpublichealth.biomedcentral.com/articles/10.1186/s12889-020-08877-1 10.1186/s12889-020-08877-1 PMC7240928 32434499

[12] Amenu K, Szonyi B, Grace D, Wieland B. Important knowledge gaps among pastoralists on causes and treatment of udder health problems in livestock in southern Ethiopia: results of qualitative investigation. BMC Vet Res [Internet]. 2017 Oct 23 [cited 2021 Mar 12];13(1):303. Available from: 10.1186/s12917-017-1222-1 10.1186/s12917-017-1222-1 PMC5651631 29058605

[13] Volpato G, Lamin Saleh SM, Di Nardo A. Ethnoveterinary of Sahrawi pastoralists of Western Sahara: camel diseases and remedies. J Ethnobiol Ethnomedicine [Internet]. 2015 Jun 20 [cited 2021 Mar 12];11(1):54. Available from: 10.1186/s13002-015-0040-4 10.1186/s13002-015-0040-4 PMC4477503 26087846

[14] Heffernan C, Misturelli F. The delivery of veterinary services to the poor: preliminary findings from Kenya. Reading, UK: Veterinary and Economics Research Unit, Department of Agriculture, University of Reading; 2000. p. 88.

[15] Seifu E, Tafesse B. Prevalence and etiology of mastitis in traditionally managed camels (*Camelus dromedarius*) in selected pastoral areas in eastern Ethiopia. Ethiop Vet J [Internet]. 2010 [cited 2020 Dec 10];14(2):103–14. Available from: https://www.ajol.info/index.php/evj/article/view/63887

[16] Ali MAI. Epidemiological study on camel mastitis in North Kordofan State, Sudan [Internet] [MSc thesis]. [Khartoum, Sudan]: University of Khartoum; 2006 [cited 2021 Mar 12]. Available from: http://khartoumspace.uofk.edu/bitstream/handle/123456789/8536/EPIDEMIOLOGICAL%20STUDY%20ON%20CAMEL%20MASTITIS.pdf?sequence=1

[17] Mengistu F, Molla P, Ali P. Camel mastitis, associated bacterial pathogens and its impact on milk quality in Gewane district, Afar regional State, Northeastern Ethiopia. Bull Anim Hlth Prod Afr. 2010;(58):241–7.

[18] Wanjohi M, Gitao CG. Bebora. Subclinical mastitis affecting hygienic quality of marketed camel milk from North-Eastern Province, Kenya. Microbiol Res Int [Internet]. 2013 Apr 10 [cited 2020 Dec 10];1(1):6–15. Available from: http://www.netjournals.org/z_MRI_13_012.html

[19] Lamuka PO, Njeruh FM, Gitao GC, Abey KA. Camel health management and pastoralists’ knowledge and information on zoonoses and food safety risks in Isiolo County, Kenya. Pastoralism [Internet]. 2017 Aug 2 [cited 2020 Dec 10];7(1):20. Available from: 10.1186/s13570-017-0095-z

[20] Chambers R. The origins and practice of participatory rural appraisal. World Dev [Internet]. 1994 Jul 1 [cited 2023 Apr 13];22(7):953–69. Available from: https://www.sciencedirect.com/science/article/pii/0305750X94901414

[21] Chambers R. Rural development: putting the last first. Vol. 1983. London: Longman; 1983.

[22] Pretty J et al. Participatory Learning and Action: a Trainer’s Guide [Internet]. Vol. 1995. London: International Institute for Environment and Development (IIED); 1995 [cited 2023 Apr 13]. Available from: https://www.iied.org/sites/default/files/pdfs/migrate/9224IIED.pdf?#page=100

[23] Catley A, Leyland T, Bishop S, Policies. Practice and participation in complex emergencies: the case of livestock interventions in South Sudan - A case study for the agriculture and development economics division of the food and agriculture organization. Boston, MA, USA: Alan Shawn Feinstein International Famine Centre School of Nutrition Science and Policy Tufts University; 2005.

[24] Chenais E, Fischer K. Increasing the Local Relevance of Epidemiological Research: Situated Knowledge of Cattle Disease Among Basongora Pastoralists in Uganda. Front Vet Sci [Internet]. 2018 [cited 2021 Apr 13];5. Available from: https://www.frontiersin.org/articles/10.3389/fvets.2018.00119/full 10.3389/fvets.2018.00119 PMC6008553 29951490

[25] Wako DD, Younan M, Tessema TS, Glücks IV, Baumann MPO. Indigenous knowledge of pastoralists on respiratory diseases of camels in northern Kenya. Prev Vet Med [Internet]. 2016 Aug 1 [cited 2021 Apr 13];130:60–6. Available from: https://www.sciencedirect.com/science/article/pii/S0167587716301386 10.1016/j.prevetmed.2016.05.008 27435647

[26] Mati BM, Muchiri JM, Njenga K, Penning de Vries F, Merrey DJ. Assessing water availability under pastoral livestock systems in drought-prone Isiolo district, Kenya. Colombo, Sri Lanka: International Water Management Institute; 2005.

[27] Isiolo county government, Department of Agriculture, Livestock & Fisheries Development. Isiolo County Sectoral Plan 2018–2027 [Internet]. Nariobi, Kenya: Department of Agriculture, Livestock & Fisheries Development, Republic of Kenya; 2019 [cited 2021 Mar 10]. Available from: http://assembly.isiolo.go.ke/wp-content/uploads/2017/08/Isiolo-County-Sectoral-Plan-2018-2027-1.pdf

[28] Megersa B, Markemann A, Angassa A, Valle Zárate A. The role of livestock diversification in ensuring household food security under a changing climate in Borana, Ethiopia. Food Secur [Internet]. 2014 Feb 1 [cited 2023 Mar 2];6(1):15–28. Available from: 10.1007/s12571-013-0314-4

[29] Muloi D, Alarcon P, Ombui J, Ngeiywa KJ, Abdullahi B, Muinde P et al. Value chain analysis and sanitary risks of the camel milk system supplying Nairobi city, Kenya. Prev Vet Med [Internet]. 2018 Nov 1 [cited 2021 Mar 2];159:203–10. Available from: https://www.sciencedirect.com/science/article/pii/S0167587718302435 10.1016/j.prevetmed.2018.09.010 PMC6193137 30314783

[30] Kagunyu AW, Wanjohi J. Camel rearing replacing cattle production among the Borana community in Isiolo County of Northern Kenya, as climate variability bites. Pastoralism [Internet]. 2014 Aug 28 [cited 2021 Feb 8];4(1):13. Available from: 10.1186/s13570-014-0013-6

[31] Kerstetter K, Insider. Outsider, or Somewhere Between: The Impact of Researchers’ Identities on the Community-Based Research Process. J Rural Soc Sci [Internet]. 2012;27(2). Available from: https://egrove.olemiss.edu/jrss/vol27/iss2/7

[32] Mburu CM, Bukachi S, Majiwa H, Ongore D, Baylis M, Mochabo K et al. Prioritization of livestock diseases by pastoralists in Oloitoktok Sub County, Kajiado County, Kenya. PLOS ONE [Internet]. 2023 Jul 12 [cited 2024 Feb 12];18(7):e0287456. Available from: https://journals.plos.org/plosone/article?id=10.1371/journal.pone.0287456 10.1371/journal.pone.0287456 PMC10337939 37436965

[33] Chenais E, Wennström P, Kartskhia N, Fischer K, Risatti G, Chaligava T et al. Perceptions of pastoralist problems: A participatory study on animal management, disease spectrum and animal health priorities of small ruminant pastoralists in Georgia. Prev Vet Med [Internet]. 2021 Aug 1 [cited 2023 Apr 13];193:105412. Available from: https://www.sciencedirect.com/science/article/pii/S0167587721001562 10.1016/j.prevetmed.2021.105412 34144495

[34] Hadef L, Hamad B, Aggad H. Risk factors associated with subclinical mastitis and its effect on physico-mineral features of camel milk. Trop Anim Health Prod [Internet]. 2022 Jul 4 [cited 2024 Apr 25];54(4):224. Available from: 10.1007/s11250-022-03220-9 10.1007/s11250-022-03220-9 35788910

[35] Waage S, Sviland S, Ødegaard SA. Identification of Risk Factors for Clinical Mastitis in Dairy Heifers. J Dairy Sci [Internet]. 1998 May 1 [cited 2024 Apr 25];81(5):1275–84. Available from: https://www.sciencedirect.com/science/article/pii/S0022030298756899 10.3168/jds.S0022-0302(98)75689-9 9621229

[36] Regassa A, Golicha G, Tesfaye D, Abunna F, Megersa B. Prevalence, risk factors, and major bacterial causes of camel mastitis in Borana Zone, Oromia Regional State, Ethiopia. Trop Anim Health Prod [Internet]. 2013 Oct 1 [cited 2020 Dec 11];45(7):1589–95. Available from: 10.1007/s11250-013-0403-6 10.1007/s11250-013-0403-6 23563738

[37] Adane B, Bayissa B, Tuffa S, Tola T, Mekonnen S. Participatory impact assessment of ticks on cattle milk production in pastoral and agro-pastoral production systems of Borana Zone, Oromia Regional State, Southern Ethiopia. Ethiop Vet J [Internet]. 2012 [cited 2023 Mar 2];16(1):1–13. Available from: https://www.ajol.info/index.php/evj/article/view/78518

[38] Pyörälä S. New Strategies to Prevent Mastitis. Reprod Domest Anim [Internet]. 2002 [cited 2021 Mar 17];37(4):211–6. Available from: https://onlinelibrary.wiley.com/doi/abs/10.1046/j.1439-0531.2002.00378.x 10.1046/j.1439-0531.2002.00378.x 12173986

[39] Barto PB, Bush LJ, Adams GD. Feeding milk containing *Staphylococcus aureus* to calves. J Dairy Sci. 1982;65(2):271–4. 7076957 10.3168/jds.S0022-0302(82)82187-5

[40] Capurro A, Aspán A, Ericsson Unnerstad H, Persson Waller K, Artursson K. Identification of potential sources of *Staphylococcus aureus* in herds with mastitis problems. J Dairy Sci [Internet]. 2010 Jan 1 [cited 2021 Feb 5];93(1):180–91. Available from: https://www.sciencedirect.com/science/article/pii/S0022030210702782 10.3168/jds.2009-2471 20059917

[41] Seligsohn D, Crestani C, Gitahi N, Lejon Flodin E, Chenais E, Zadoks RN. Investigation of extramammary sources of Group B *Streptococcus* reveals its unusual ecology and epidemiology in camels. PLoS ONE [Internet]. 2021 Dec 3 [cited 2024 Apr 25];16(12):e0252973. Available from: https://www.ncbi.nlm.nih.gov/pmc/articles/PMC8641886/10.1371/journal.pone.0252973 PMC8641886 34860840

[42] Chenais E, Fischer K, Aliro T, Ståhl K, Lewerin SS. Co-created community contracts support biosecurity changes in a region where African swine fever is endemic– Part II: Implementation of biosecurity measures. Prev Vet Med [Internet]. 2023 May 1 [cited 2023 Apr 13];214:105902. Available from: https://www.sciencedirect.com/science/article/pii/S0167587723000661 10.1016/j.prevetmed.2023.105902 36966659

[43] Alemu B, Desta H, Kinati W, Mulema AA, Gizaw S, Wieland B. Application of Mixed Methods to Identify Small Ruminant Disease Priorities in Ethiopia. Front Vet Sci [Internet]. 2019 Nov 26 [cited 2024 May 7];6. Available from: https://www.frontiersin.org/articles/10.3389/fvets.2019.00417 10.3389/fvets.2019.00417 PMC6988793 32039243

[44] Simpkins SP. The effects of breed and management on milk yield of camels in Kenya [Ph.D.Thesis]. [UK,]: University of Newcastle; 1996.

[45] Griffith E, Kipkemoi JR, Mariner JC, Coffin-Schmitt J, Whittier CA. Expanding Participatory Epidemiology to Explore Community Perceptions of Human and Livestock Diseases among Pastoralists in Turkana County, Kenya [Internet]. 2023 [cited 2024 May 7]. Available from: https://www.qeios.com/read/AKQZXP

[46] Dodd FH, Westgarth DR, Neave FK, Kingwill RG. Mastitis—The Strategy of Control. J Dairy Sci [Internet]. 1969 May 1 [cited 2021 Mar 17];52(5):689–95. Available from: https://www.sciencedirect.com/science/article/pii/S0022030269866312 10.3168/jds.S0022-0302(69)86631-2 5391702

[47] Kena D. Review on camel production and marketing status in Ethiopia. Pastoralism [Internet]. 2022 Sep 12 [cited 2024 Jun 18];12(1):38. Available from: 10.1186/s13570-022-00248-2 10.1186/s13570-022-00248-2 PMC9465664 36117775

[48] Amenu K, Agga GE, Kumbe A, Shibiru A, Desta H, Tiki W et al. *MILK Symposium review*: Community-tailored training to improve the knowledge, attitudes, and practices of women regarding hygienic milk production and handling in Borana pastoral area of southern Ethiopia*. J Dairy Sci [Internet]. 2020 Nov 1 [cited 2024 Jun 18];103(11):9748–57. Available from: https://www.sciencedirect.com/science/article/pii/S0022030220307803 10.3168/jds.2020-18292 33076186

[49] Valeeva NI, Lam TJGM, Hogeveen H. Motivation of Dairy Farmers to Improve Mastitis Management. J Dairy Sci [Internet]. 2007 Sep 1 [cited 2024 May 15];90(9):4466–77. Available from: https://www.sciencedirect.com/science/article/pii/S0022030207719094 10.3168/jds.2007-0095 17699068

[50] Kaufmann B. Differences in perception of causes of camel calf losses between pastoralists and scientists. Exp Agric. 2003;39:363–78.

[51] Saleh MS, Mobarak AM, Fouad SM, Radiological. Anatomical and Histological Studies of the Mammary Gland of the One-humped Camel (*Camelus Dromedarius*). Zentralblatt Für Veterinärmedizin Reihe A [Internet]. 1971 [cited 2021 Mar 5];18(4):347–52. Available from: https://onlinelibrary.wiley.com/doi/abs/10.1111/j.1439-0442.1971.tb00587.x 10.1111/j.1439-0442.1971.tb00587.x 4998115

[52] Petrovski KR, Eats PT. Best practice: why mastitis treatment is not successful? In Perth, Australia: Australian Veterinary Association; 2014.

[53] Fischer A, Liljander A, Kaspar H, Muriuki C, Fuxelius HH, Bongcam-Rudloff E et al. Camel *Streptococcus agalactiae* populations are associated with specific disease complexes and acquired the tetracycline resistance gene tetM via a Tn916-like element. Vet Res [Internet]. 2013 Oct 1 [cited 2020 Dec 10];44(1):86. Available from: 10.1186/1297-9716-44-86 10.1186/1297-9716-44-86 PMC3850529 24083845

[54] Seligsohn D, Crestani C, Forde TL, Chenais E, Zadoks RN. Genomic analysis of group B *Streptococcus* from milk demonstrates the need for improved biosecurity: a cross-sectional study of pastoralist camels in Kenya. 2021. 10.1186/s12866-021-02228-9 PMC8287776 34281509

[55] Fang W, Pyörälä S. Mastitis-causing *Escherichia coli*: serum sensitivity and susceptibility to selected antibacterials in milk. J Dairy Sci. 1996;79(1):76–82. 8675785 10.3168/jds.S0022-0302(96)76336-1

[56] Kuang Y, Jia H, Miyanaga K, Tanji Y. Effect of milk on antibacterial activity of Tetracycline against *Escherichia coli* and *Staphylococcus aureus* isolated from bovine mastitis. Appl Microbiol Biotechnol. 2009;84(1):135–42. 19418049 10.1007/s00253-009-2008-6

[57] Gruet P, Maincent P, Berthelot X, Kaltsatos V. Bovine mastitis and intramammary drug delivery: review and perspectives. Adv Drug Deliv Rev [Internet]. 2001 [cited 2021 Mar 11];50(3):245–59. Available from: https://www.sciencedirect.com/science/article/pii/S0169409X01001600 10.1016/s0169-409x(01)00160-0 11500230

[58] Lents CA, Wettemann RP, Paape MJ, Vizcarra JA, Looper ML, Buchanan DS, et al. Efficacy of intramuscular treatment of beef cows with Oxytetracycline to reduce mastitis and to increase calf growth. J Anim Sci. 2002;80(6):1405–12. 12078719 10.2527/2002.8061405x

[59] Nielsen C, Emanuelson U. Mastitis control in Swedish dairy herds. J Dairy Sci [Internet]. 2013 Nov 1 [cited 2021 Apr 8];96(11):6883–93. Available from: https://www.sciencedirect.com/science/article/pii/S0022030213006280 10.3168/jds.2012-6026 24054281

[60] Musinga M, Kimenye D, Kivolonzi P. The camel milk industry in Kenya. Nairobi, Kenya: Netherlands Development Organisation (SNV);: Resource Mobilization Centre; 2008.

[61] Odongo N, Matofari J, Abong G, Lamuka P, Abey K. Knowledge and practices of food hygiene and safety among camel milk handlers in the pastoral camel value chain in Kenya. Afr J Food Agric Nutr Dev. 2017;17:11803–21.

[62] Ahmad S, Yaqoob M, Bilal MQ, Muhammad G, Yang LG, Khan MK et al. Risk factors associated with prevalence and major bacterial causes of mastitis in dromedary camels (*Camelus dromedarius*) under different production systems. Trop Anim Health Prod [Internet]. 2012 Jan 1 [cited 2020 Dec 10];44(1):107–12. Available from: 10.1007/s11250-011-9895-0 10.1007/s11250-011-9895-0 21660648

[63] Lunner-Kolstrup C, Ssali TK. Awareness and Need for Knowledge of Health and Safety among Dairy Farmers Interviewed in Uganda. Front Public Health [Internet]. 2016 Jun 28 [cited 2024 Jun 19];4. Available from: https://www.frontiersin.org/journals/public-health/articles/10.3389/fpubh.2016.00137/full 10.3389/fpubh.2016.00137 PMC4923150 27446901

[64] Lunner Kolstrup C, Hultgren J. Perceived Physical and Psychosocial Exposure and Health Symptoms of Dairy Farm Staff and Possible Associations with Dairy Cow Health. J Agric Saf Health [Internet]. 2011 [cited 2024 Jun 19];17(2):111–25. Available from: 10.13031/2013.36496 10.13031/2013.36496 21675282

[65] Belage E, Croyle SL, Jones-Bitton A, Dufour S, Kelton DF. A qualitative study of Ontario dairy farmer attitudes and perceptions toward implementing recommended milking practices. J Dairy Sci [Internet]. 2019 Oct 1 [cited 2024 May 15];102(10):9548–57. Available from: https://www.sciencedirect.com/science/article/pii/S002203021930606X 10.3168/jds.2018-15677 31326172

