# Supplementary material for: Camel pastoralists’ perceptions of udder health: results from a qualitative interview study in Northern Kenya
Source: BMC Vet Res. 2025 Apr 14;21:270. doi: 10.1186/s12917-025-04723-x (PMC11995632; doi:10.1186/s12917-025-04723-x)
Supplement: Supplementary file 1 — Supplementary material 1: Topic guide [file 12917_2025_4723_MOESM1_ESM.docx]

# Camel mastitis, Northern Kenya

## Focus group discussions

1. Present the project and its objectives and the project team
2. Explain the consent form and the confidentiality
3. Fill in background data sheet
4. Start the discussion

## Interview/topic guide

### Warm up

1. Do you own/look after camels? (give everybody a chance to share their answer)
2. Can you describe how you manage your camels?

### Disease perception

1. How would you describe a healthy camel?
2. Does it happen that you have problems with disease in your camels?
3. Which diseases have you experienced in your herd that you can remember/or decide in the past 2 years? (List diseases on flip chart)
4. Which ones are more important and why? (Rank the 5 most important ones. For each: Describe the signs and discuss why this disease is important.

### Disease management

1. What do you do to keep your camels healthy?
2. What do you do when your camels are not healthy?

### Mastitis

1. Do your camels have problems with their udders?
2. What kind of udder problems are there? (List all problems, for each discuss:)
3. Describe the symptoms?
4. What happens? (describe the process)
5. What causes this udder problem?

### Mastitis management

1. How do you protect your animals from udder problems?
2. How do you treat udder problems?
3. What do you do with the milk from unhealthy udders?
4. What other actions do you think could help the udder health?

### Present and discuss the following possible control measures:

1. Milking order – milking the oldest and sickest camels last.
2. Hand hygiene – using disposable gloves that are changed or hand disinfectant between every camel that is milked.
3. Udder hygiene – wipe the udder with wet tissues before milking
4. CMT – California Mastitis Test – Testing the milk of the camels to find out which one has an infection in the udder.
5. Teat dipping - disinfecting the teats at the end of each milking.

### Rounding up

1. Anything else you would like to discuss?
